# Differences in Endothelin B Receptor Isoforms Expression and Function in Breast Cancer Cells

**DOI:** 10.7150/jca.41004

**Published:** 2020-02-19

**Authors:** Meena Halaka, Zuhaila A. Hired, Grace E. Rutledge, Carly M. Hedgepath, Michael P. Anderson, Haley St. John, Jessica M. Do, Parth R. Majmudar, Caleb Walker, Asma Alawawdeh, Hannah M. Stephen, Caleb C. Reagor, Jeanette Adereti, Kiara Jamison, Katherine P. Iglesias, Khadija Z. Kirmani, Rebecca E. Conway

**Affiliations:** Department of Biology, College of Liberal Arts and Sciences, Lipscomb University, 1 University Park Drive, Nashville, TN 37204, USA

## Abstract

The endothelins and their receptors are best known for their regulation of the vascular system. Their widespread expression in epithelial cells and their overexpression in some tumors has prompted investigation into their ability to regulate cancer progression. In this study, we assessed the mRNA expression of the major endothelin B receptor gene (EDNRB) isoforms and found differences in both mRNA and protein expression in normal breast cells and breast cancer cell lines. Knocking down the EDNRB gene in breast cancer cells altered invasiveness toward endothelin 3 (ET3), and we observed EDNRB isoform-specific regulation of breast cancer cell invasion and cell signaling, as well as isoform- and subtype-specific differences in breast cancer patient survival. The results reported in this study emphasize the importance of the endothelin B receptor in breast cancer. To our knowledge, this study is the first to clarify the differential expression and roles of specific EDNRB isoforms in breast cancer.

## Introduction

The Endothelin Axis is comprised of the endothelin (ET) peptides ET1-3, the endothelin A receptor and endothelin B receptor (EDNRA and EDNRB, respectively) and endothelin converting enzyme (ECE); this axis is well-characterized in various tissues and diseases (reviewed in 1). The endothelin receptors are both G-protein coupled receptors (GPCRs); while EDNRA associates with G_q_ and G_s_, EDNRB associates with G_q_ and G_i_ 2,3. Cell survival, proliferation, and migration are stimulated in the presence of endothelins and are dependent on endothelin receptor activation of the mitogen activated protein kinase pathway (MAPK) and the phosphoinositide-3 kinase (PI3K) pathway (reviewed in 1). The endothelin peptides bind to their receptors, EDNRA and EDNRB with differing affinities. While EDNRA preferentially binds ET1 and ET2, EDNRB binds to ET1, ET2, and ET3 with equal affinity 4. Additionally, EDNRB has been shown to internalize ET1, suggesting it may play a role in negatively regulating endothelin signaling 5.

Because the endothelin axis is best characterized in the vasculature, studies of endothelins and their receptors in the vascular system may provide insight into the endothelin axis in other tissues. Numerous studies report differences in EDNRA and EDNRB internalization following ligand binding; while EDNRA is recycled back to the plasma membrane following ET1 binding and internalization, EDNRB is targeted to the lysosomal pathway 6, 7, 8; in this context, EDNRB is believed to function as a “clearance receptor” for endothelins 9, removing ET1 from circulation 9,10. Furthermore, while multiple studies demonstrate activating effects of ET1/ endothelin receptor binding^11^, a recent study found that in rat coronary arteries, high ET3 levels inhibited activation of EDNRB and endothelin signaling. Together, these data suggest that in the vascular system the endothelins and their receptors appear to have distinct roles, and EDNRB may act as negative regulator of endothelin signaling, while ET1 and EDNRA promote endothelin signaling. Whether these distinct roles apply to other tissues and disease contexts remains unclear.

The endothelin axis has been extensively studied in multiple cancer types including breast cancer, yet important questions remain unanswered (reviewed in 1). In both clinical breast cancer samples and breast cancer cell lines, endothelins and endothelin A receptor expression correlate with increased vascularization and invasion and decreased survival 12,13, 14,15,16,17,18, consistent with its reported role in other cancers. Furthermore, chemically inhibiting EDNRA inhibits invasion in breast cancer cell lines 19, and ET1 and ET2 both induce breast cancer cell migration in an EDNRA and EDNRB-dependent manner 16,20. In contrast, the effects of ET3 and its selective binding to EDNRB on endothelin signaling and cancer progression may be dependent on cancer type. For example, ET3 expression is suppressed in breast, colon cancer and cervical cancer 21, 22, 23, 24, suggesting an inhibitory role of ET3/EDNRB signaling in these cancers. In melanoma however, ET3 increases cancer cell migration and survival 25,26, 27,28,29. The precise role of the ET3-activated endothelin B receptor (EDNRB) signaling in cancer remains unclear, and the effects of ET3-stimulated EDNRB in breast cancer are not fully understood. Another complexity of endothelin signaling involve the multiple EDNRB isoforms that are predicted to encode for functional G-protein coupled receptors (GPCRs). Isoform expression differences between normal cells and cancer cells, and potential functional differences of these EDNRB isoforms in cancer have not been previously reported in breast cancer.

**Figure 1:**
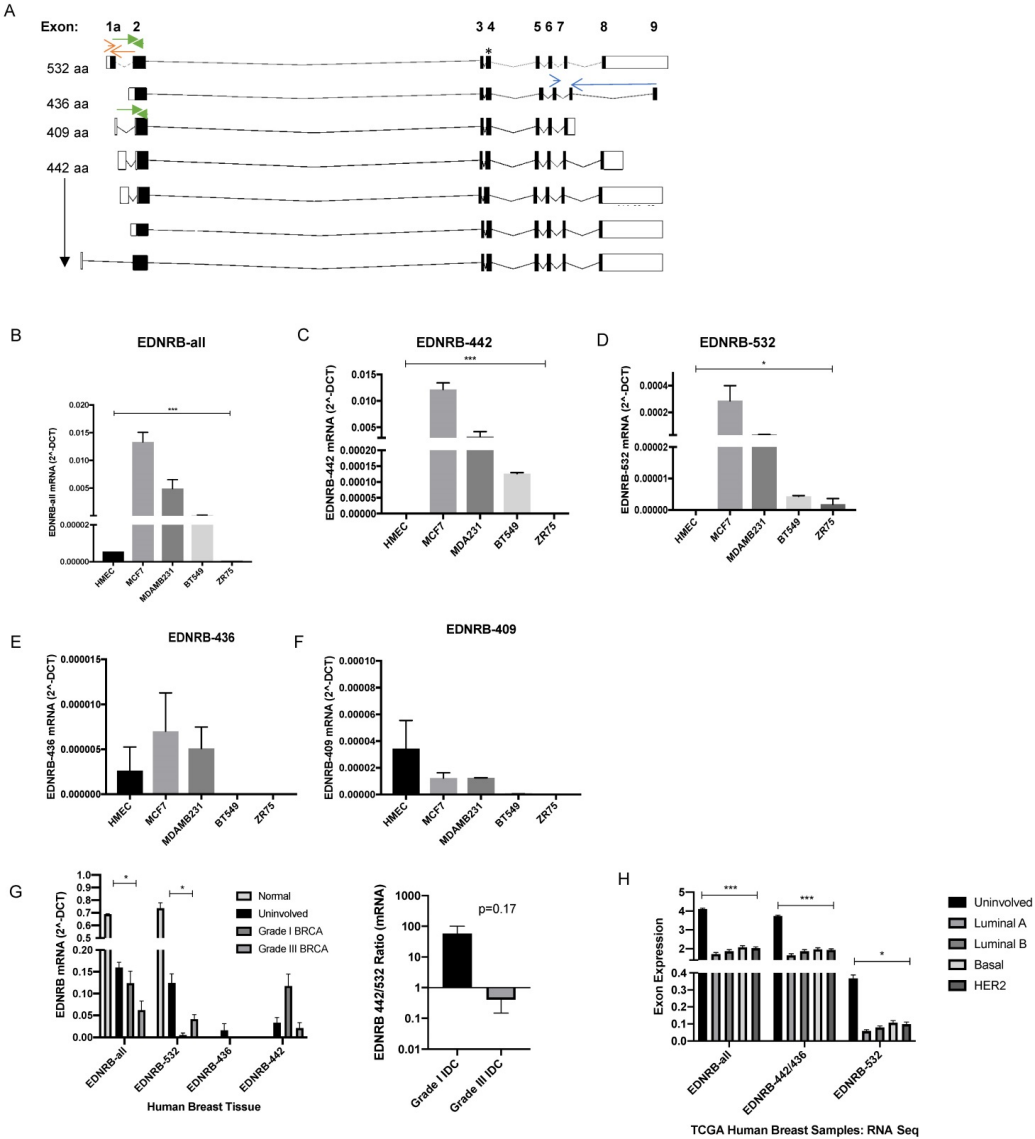
** EDNRB mRNA expression in breast cancer cell lines.** (A) Schematic of ENDRB splice variants; open boxes are non-coding exon sequences and closed boxes indicate coding exon sequences. * indicates the location of the EDNRB-all primers; red arrows indicate the location of the EDNRB-532 primers; blue arrows indicate the location of the EDNRB-436 primers; green arrows represent the location of the EDNRB-532/409 primers. (B)-(F) Semi-quantitative RT-PCR results from EDNRB-all primers tested with pooled biological triplicates from HMEC, MDA-MB-231, MCF7, BT-549, and ZR-75 cell with (B) EDNRB-all primers (ANOVA p<0.0001); (C) EDNRB-442 primers (ANOVA p=0.0003); (D) EDNRB-532 primers (ANOVA p=0.028); (E) EDNRB-436 primers (ns); and (F) deduced EDNRB-409 (see methods; ns). (G) Semi-quantitative RT-PCR results from EDNRB isoform-specific primers with RNA extracted from uninvolved/normal breast tissue and grade III metastatic invasive ductal carcinoma (n=3); EDNRB-all p=0.015; EDNRB-532 p=0.024; *right-* The ratio of EDNRB-442/532 from RTPCR analysis shows differences in low-grade vs high-grade IDC. (H) TCGA dataset analysis of EDNRB isoform expression by breast cancer subtype and normal tissue. (2-way ANOVA p<0.0001; Tukey's comparison shows significance between normal and all subtypes for EDNRB-all and EDNRB-436/442 p<0.0001; normal vs all subtypes for EDNRB-532 p≤0.01).

In the present study, we focused on characterizing the expression of function of EDNRB isoforms in breast cancer cells to more clearly understand the role of EDNRB in breast cancer. The EDNRB gene is alternatively spliced into multiple isoforms with unique amino acid sequences. All major variants encode for 7-transmembrane domain G-protein coupled receptors, yet to our knowledge the individual expression and contribution of these isoforms to breast cancer cells has not been reported. Currently, 8 distinct transcript variants have been reported for EDNRB 30. Four different alternatively spliced variants all encode for the same 442-amino acid canonical isoform; addition of a 5' exon results in a 532-amino acid isoform, while an alternative 3' exon is incorporated into the 436-amino acid isoform. A 409-amino acid isoform lacks a 3' terminal exon, and a 162-amino acid isoform does not encode for a transmembrane protein and was therefore not further studied here (Fig 1A), 30. In this study, we report for the first time that EDNRB isoforms are differentially expressed across multiple breast cancer cell lines. Importantly, we also report that EDNRB-442 is the primary isoform responsible for ET3-induced inhibition of cancer cell invasion and activation of pAKT1, while EDNRB-532 promotes cell viability in some breast cancer cell lines. Together, these results establish novel isoform-specific roles for EDNRB in breast cancer cells.

## Materials and Methods

### Cell Culture

MCF-7, ZR-75, BT-549, MDA-MB-231, and HEPG2 cells were obtained from ATCC (ATCC, Manassas, VA). MCF-7 and BT-549 cells cultures were initiated in Eagle's Minimum Essential Medium with 10% fetal bovine serum and 10 μg/ml insulin. MDA-MB-231 and ZR75 cells were cultured in Dulbecco's Modified Eagle's Medium (DMEM) supplemented with 10% fetal bovine serum. Human Mammary Epithelial Cells (HMECs) were obtained from Lonza (Lonza Group, Ltd, Basel, Switzerland) and cultured with the recommended Mammary Epithelial Cell Growth Medium supplemented with the MEGM Bulletkit. All cells were cultured in DMEM supplemented with 10% FBS for at least 48 hours before RNA or protein analysis or assay set-up. Cells were grown at 37°C and 5% CO_2_ and were used within 10 passages after receipt.

### Invasion Assays

Matrigel (Becton Dickinson, San Jose, CA) was diluted 1:5 with serum-free DMEM and coated on 24-well FluoroBlok invasion inserts (Corning, Inc, Corning, NY) and incubated at 37°C for 1 hour. Cells were detached from culture dishes using trypsin-EDTA, washed two times with 1x PBS, counted, and resuspended to 100,000 cells/mL in serum-free DMEM. 100 μL of cell suspension was pipetted into the top chamber, and 600 μL of complete growth media containing 10% FBS was pipetted into the lower chamber. Plates were covered with lids and incubated at 37°C for 16 hours. After removal of cells from the top chamber by pipetting, inserts were incubated in 600 μL of PBS/calcein AM (Thermo Fisher Scientific, Waltham, MA) for 30 minutes and imaged using a Cytation3 Inverted fluorescent microscope/plate reader (BioTek, Winooski, VT). Cell counts were generated using the accompanying Cytation3 software.

### Chemicals, Nucleic Acids, Plasmids, and Antibodies

Endothelin 3 was purchased from Sigma Aldrich and reconstituted to 1 mg/ml in water. siRNA specific for EDNRB (sc-39962), along with control siRNA (sc-37007) were purchased from Santa Cruz (Santa Cruz Biotechnology, Santa Cruz, CA). EDNRB antibodies for Western blotting (ab117529) and flow cytometry (ab129102) and GAPDH antibody for Western blotting (ab9485) 31 were purchased from Abcam (Cambridge, United Kingdom). pERK (#4370) and ERK (#4695) 32 antibodies, along with pan AKT (4691), phospho AKT T308 (13038) and phospho-AKT1 S473 and phospho-AKT2 S474 (8599) were all purchased from Cell Signaling (Cell Signaling Technology, Beverly, MA). Secondary antibodies, including goat anti-mouse IgG-HRP, goat anti-rabbit IgG-HRP, goat anti-rabbit IgG-FITC were all purchased from Cell Signaling. Plasmid expression vectors encoding EDNRB-442 (RG 232943), EDNRB-536 (RC 216462), and EDNRB-436 (RC 225723) and accompanying empty vector controls (pCMV6-Entry vector) were purchased from Origene (Rockville, MD).

### Cell Transfections

For siRNA transfections, MCF-7 or MDA-MB-231 cells were plated in 6-well plates at 50% confluency 12 hours prior to transfection in complete growth media in the absence of antibiotic. 100 pmol per well of gene-specific or scrambled siRNA was diluted in RNAi Max (Thermo Fisher Scientific) according to the manufacturer's protocol. 72 hours following transfection, cells were harvested for RNA extraction or invasion assays. For transfecting expression plasmids, 2.5 μg of empty vector or plasmid DNA was diluted with Lipofectamine 3000 (Thermo Fisher Scientific) according to the manufacturer's protocol and added to cells grown in 6-well plates. 72 hours following transfection, cells were analyzed for RNA, protein, or cell function based on the described assays.

### Western Blotting

For EDNRB-isoform Western blots, cells were transfected with empty vector or EDNRB-(442/436/532) expression vectors as described above 72 hours prior to protein extraction. 2 hours prior to protein extraction, cells were cultured in serum-free DMEM and 1 hour prior to protein extraction, cells were stimulated with 100 nM ET-3. For Western blots, Halt Protease Inhibitor Cocktail (Life Technologies) was added to RIPA buffer according to the manufacturer's protocol. For phosphorylated proteins, we added protease and phosphatase inhibitor (Sigma Aldrich). Growth media was aspirated, and cells were washed in PBS twice. 1 mL of RIPA buffer per 5x10^6^ cells was added to cell culture plates and incubated on ice for 5 minutes. Cells were scraped and transferred to a tube on ice, then centrifuged at 14,000x g for 15 minutes. Supernatant was transferred to a new tube and stored at -80°C until used for Westerns. Equal amounts of protein lysate were mixed with Laemmli Sample Buffer (Bio-Rad, Hercules, CA), incubated at room temperature for 30 minutes (for EDNRB) or boiled for 5 minutes and loaded onto Mini-PROTEAN TGX Gels, 4-20% (Bio-Rad). Gels were transferred to nitrocellulose membranes (Bio-Rad). Western blotting was performed using the Opti-4CN Substrate Kit (Bio-Rad) following the manufacturer's protocol at the recommended dilutions for each antibody. Membranes were blocked at room temperature for 1 hour, incubated with primary antibody at 4°C overnight, and incubated with secondary antibody (1:2000 for goat anti-rabbit IgG HRP and 1:3000 for goat anti-mouse IgG HRP) for 1 hour at room temperature with shaking. Colorimetric detection was performed using the Opti-4CN substrate and Amplification kit (Bio-Rad), blots were imaged on the Bio-Rad Gel Doc XR System, and bands were quantified using ImageJ software 33 and normalized to GAPDH band intensity. All blots from the same replicate were processed in parallel.

### RNA extraction from clinical tumor samples

Tumors representing invasive ductal carcinoma and matched adjacent normal were provided by the Cooperative Human Tissue Network (CHTN), a National Cancer Institute supported resource. Other investigators may have received samples from these same tissue specimens. Tumor samples were anonymously coded by the CHTN, and the protocol was approved and exempt-status was granted by Lipscomb University's Institutional Review Board. RNA extraction was performed on ≤150mg tissue using the Quick RNA MiniPrep Kit according to the manufacturer's protocol (Zymo Research, Irvine, CA).

### RT-qPCR

RNA was isolated from cultured cells using Qiagen's RNeasy kit according to the manufacturer's protocol (Qiagen Inc, Valencia, CA). Following RNA extraction, RNA concentration and purity were measured using a TECAN plate reader (San Jose, CA). 0.5 μg of RNA was reverse transcribed using the Bio-Rad iScript cDNA synthesis kit according to the manufacturer's recommended protocol (Bio-Rad Laboratories, Inc, Hercules, CA). The thermocycler conditions were as follows: 94 °C-3 min, followed by 40 cycles of 94 °C-15 s, 57.5 °C-45 s, 72 °C-45 s, followed by a melt curve analysis. Analysis was performed using the ΔΔCT method 34; statistical analysis was conducted on 2^-DCt^ values. Analysis was performed over three independent experiments with technical replicates; All primers used for qPCR are listed, along with their sequences, in Supplementary Table 1. While we were able to design primers to specifically detect EDNRB-436 and EDNRB-532, the shared exons of EDNRB-442 and EDNRB-409 required us to deduce relative expression using combinations of primers. Amplification with primers common to all major EDNRB isoforms (EDNRB-all), EDNRB-532, and EDNRB-436 primers were compared to deduce EDNRB-442 according to the formula:

EDNRB-442= 2^-DCT (all EDNRB)- 2^-DCT (EDNRB-532)- 2^-DCT (EDNRB-436)

EDNRB-409=2^-DCT (EDNRB-532/409) -2^-DCT(EDNRB-532).

### Flow cytometry

Cells were detached from cell culture dishes, rinsed in PBS, centrifuged, and counted. Cells were washed in BD stain buffer (Becton Dickinson) and resuspended in BD Stain Buffer with primary antibody (1:250 for ETBR, 1:40 for ETAR, 10 μg/1x10^5^ cells for ECE1**)** to a concentration of 1x10^5^ cells/100 μl and incubated at room temperature for 45 minutes. Cells were washed twice with BD Stain Buffer and re-suspended in secondary antibody diluted in BD Stain Buffer (1:100) and incubated at room temperature for 45 minutes. Cells were washed twice and re-suspended in 200 μl BD Stain Buffer for flow cytometry analysis. Cells were analyzed on BD Accuri C6 flow cytometer and BD CSampler software (Becton, Dickinson and Company, Franklin Lakes, NJ).

### MTT assays

Cells were transfected with empty vector or EDNRB plasmids as described above. 24 hours after transfection, cells were trypsinized, counted, and seeded at 20% confluency in 96-well plates. Cells were allowed to fully adhere, and day 0 plates were treated with MTT substrate using the MTT Cell Proliferation Assay kit (ATCC) according to the manufacturer's protocol. Absorbance was ready at 570 nm and 670 nm (reference), and the blank-adjusted difference between 570 nm and the reference wavelength was calculated. Values were normalized to empty vector controls from the same plate. This was repeated at 24 hour, 48 hours, and 72 hours after trypsinizing and seeding cells. All experiments were conducted in technical triplicate and biological replicate.

### Bioinformatics

Clinical, RNAseq, and RPPA datasets from The Cancer Genome Atlas Breast Cancer (TCGA BRCA) database, TCGA ovarian cancer database, TCGA renal cell carcinoma database, and TCGA liver cancer database and the Cancer Cell Line Encyclopedia were downloaded (Xena Browser, University of California, Santa Cruz, xena.ucsc.edu). Data was sorted into high- and low- expression groups by the mean expression for each gene for each cancer (and for breast cancer by subtype). Survival curves were constructed using GraphPad Prism software (Graph Pad, La Jolla, CA) and analyzed by Mantel-Cox curve comparison. Exon-specific TCGA expression data was used to deduce the relative expression of specific EDNRB isoforms as described below (see Supplementary Table 2 for probe/exon location information). Because the available probes cannot fully differentiate between all EDNRB-442 encoding variants and EDNRB-436, we combined these isoforms for survival analysis (see Supplementary Table 2 for exon locations of probes).

EDNRB-532= expression of probe 8

EDNRB-442/436= (mean: expression of probes 2/3/4/5/6) - expression probe 8

### Statistical Analysis

All experiments were performed in at least technical duplicate and biological triplicate. Data was normalized where appropriate as described in the text. Technical replicates from a single biological replicate were averaged, and independent biological replicates were analyzed for statistical significance. For comparison of 2 data sets, unpaired t-tests were used, and for comparison of more than 2 data sets, ANOVA and Tukey's pairwise comparisons were used. The Mantel-Cox test was used for survival curves. Probabilities equal to or less than 0.05 were considered statistically significant. All graphs represent mean values +/- standard error.

### Data Availability Statement

The datasets analyzed during the current study were downloaded from Xena Browser (xenabrowser.net) and merged to compare RNAseq data, clinical data (breast cancer subtype and survival) and Reverse Phase Protein Array (RPPA) datasets. Merged datasets will be made available to anyone upon reasonable request.

## Results

### Endothelin B Receptor isoform expression in breast cancer cells

Previous studies have examined EDNRB mRNA and protein expression in breast cancer cell lines, but to our knowledge, expression analysis of specific EDNRB isoforms has not been previously reported in breast cancer. The eight currently reported alternatively spliced EDNRB variants 30 encode for 4 unique G-protein coupled receptors, EDNRB-442, EDNRB-532, EDNRB-436, and EDNRB-409. We designed primers spanning various isoform-specific exons in the EDNRB gene (Fig. 1A; Supplementary Table 1) to determine the relative abundance of the major EDNRB isoforms in breast cancer cell lines and normal mammary epithelial cells (HMECs). First, we used a primer pair detecting all 7 of the major GPCR-encoding isoforms (EDNRB-all) to amplify cDNA from breast cancer cells. These experiments revealed very low levels of EDNRB mRNA in the normal mammary cells (HMEC) and the ZR75 cancer cell line; the highest EDNRB expression was detected in MCF-7 cells, followed by MDA-MB-231 and BT-549 cell lines, respectively (Fig. 1B).

To assess whether the expression of specific EDNRB isoforms differed between the cell lines, we designed primers that specifically detect EDNRB-532, EDNRB-436, and a set of primers that detect both EDNRB-532 and EDNRB-409. The 442-amino acid isoform cannot be easily differentiated from the other isoforms due to the shared exons of these isoforms with nearly all other variants (Fig. 1A). Instead, to deduce the approximate EDNRB-442 expression levels, we compared the amplification using EDNRB-all primers with primers that amplified EDNRB-436 and EDNRB-532/409 (see methods for a detailed explanation). We found that EDNRB-442 is likely the most abundant isoform expressed in MCF-7, MDA-MB-231, and BT-549 cells, consistent with EDNRB-442 being the canonical isoform. This isoform was significantly more highly expressed in MCF-7 cells than all other tested cells, and was not detected in HMEC or ZR75 cells (Fig. 1C). The next most abundant isoform expressed was EDNRB-532; MCF-7 cells expressed the highest level of this isoform, and this isoform was not detected in HMECs (Fig. 1D). Analyzing expression with primers specific to the EDNRB-436 and EDNRB-409 isoforms revealed low levels across all cells, and no significant differences between cell lines (Fig. 1E-1F). To determine if these trends were specific to breast cancer, we also analyzed EDNRB isoform expression by qPCR in HEPG2 liver cancer cell line-derived cDNA; overall EDNRB expression was substantially lower in these cell lines compared to the breast cancer cell lines, and the major isoform appeared to be EDNRB-532 (Supplementary Fig S1).

To validate our cell line findings, we next analyzed RNA extracted from human clinical breast tumor samples we obtained from the Cooperative Human Tissue Network (CHTN). Normal breast tissue and uninvolved breast tissue from breast cancer patients showed higher expression than cancer samples when amplified with primers recognizing all EDNRB isoforms and with EDNRB-532 specific primers (Fig. 1G). In contrast to the results with breast cancer cell lines, we observed a trend of higher EDNRB-532 and lower EDNRB-442 in grade III compared to grade I breast cancer samples (Fig. 1G), suggesting involvement of stromal cells in EDNRB isoform expression and implicating EDNRB-532 as an isoform associated with advanced disease. Analyzing The Cancer Genome Atlas (TCGA) RNASeq exon expression database to compare exons specific for each EDNRB-isoform across breast cancer subtypes revealed no significant differences between subtypes, but all EDNRB isoforms were expressed at significantly higher levels in uninvolved breast tissue compared to breast cancer tissue, in agreement with our breast tumor EDNRB isoform expression results (Fig. 1H). Together, these results demonstrate difference in EDNRB isoform expression in breast cancer cell lines. Differences in breast cancer cell line expression and breast tumor expression may indicate the involvement of stromal cells in EDNRB isoform expression.

### Endothelin B Receptor protein expression in breast cells

**Figure 2:**
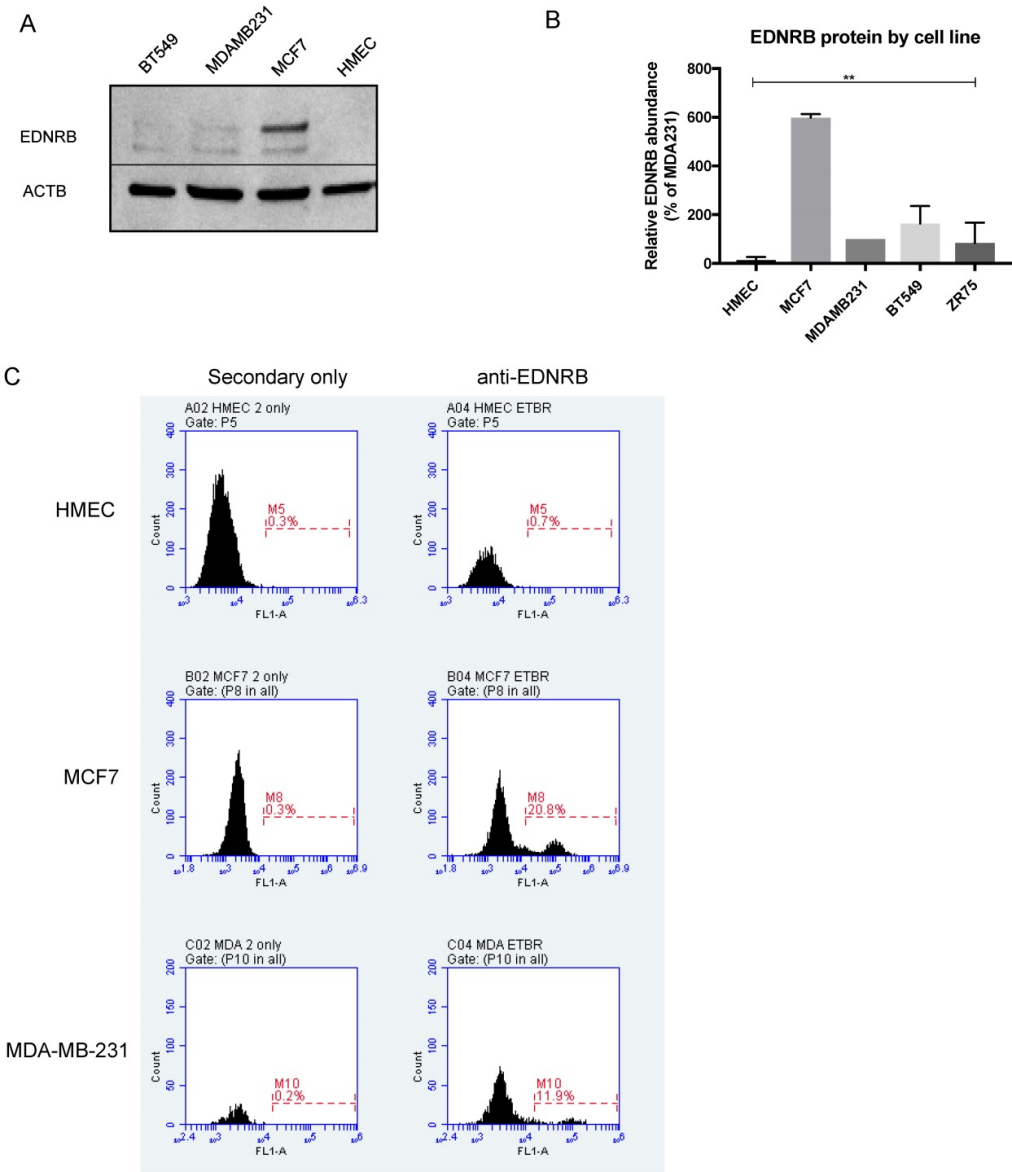
** EDNRB protein expression in breast cancer cell lines.** (A) Representative Western blot showing EDNRB protein expression in HMEC, MCF-7, MDA-MB-231 and BT-549 cell lines; (B) Semi-quantitative analysis of EDNRB relative to beta-actin (ACTB) in 3 biological replicates (2-way ANOVA p<0.0001); (C) representative flow cytometry charts showing EDNRB cell-surface expression in HMECs (top), MCF7 (middle), and MDA-MB-231 (bottom) cell lines.

We next assessed EDNRB protein expression in normal and breast cancer cell lines by Western blotting. The EDNRB-specific antibody used in these experiments detects a C-terminal amino acid sequence present only in the 532-amino acid and 442-amino acid variants. Interestingly, while little to no EDNRB protein was detected in primary HMECs, MCF7 cells expressed significantly higher overall levels of EDNRB protein compared to the other cells (Fig. 2B). In agreement with this, flow cytometry analysis revealed higher cell-surface EDNRB protein on MCF-7 cells than MDA-MB-231 cells, and HMEC cells expressed no detectable EDNRB (Fig. 2C). We also compared the expression of EDNRB in HMEC, MCF-7, and MDA-MB-231 cells using a different EDNRB antibody that recognizes all full-length isoforms; we observed a similar expression pattern, with MCF-7 cells expressing the highest level of EDNRB (Supplementary Fig. S2).

### Endothelin B Receptor isoforms differentially regulate breast cancer invasion and viability

**Figure 3:**
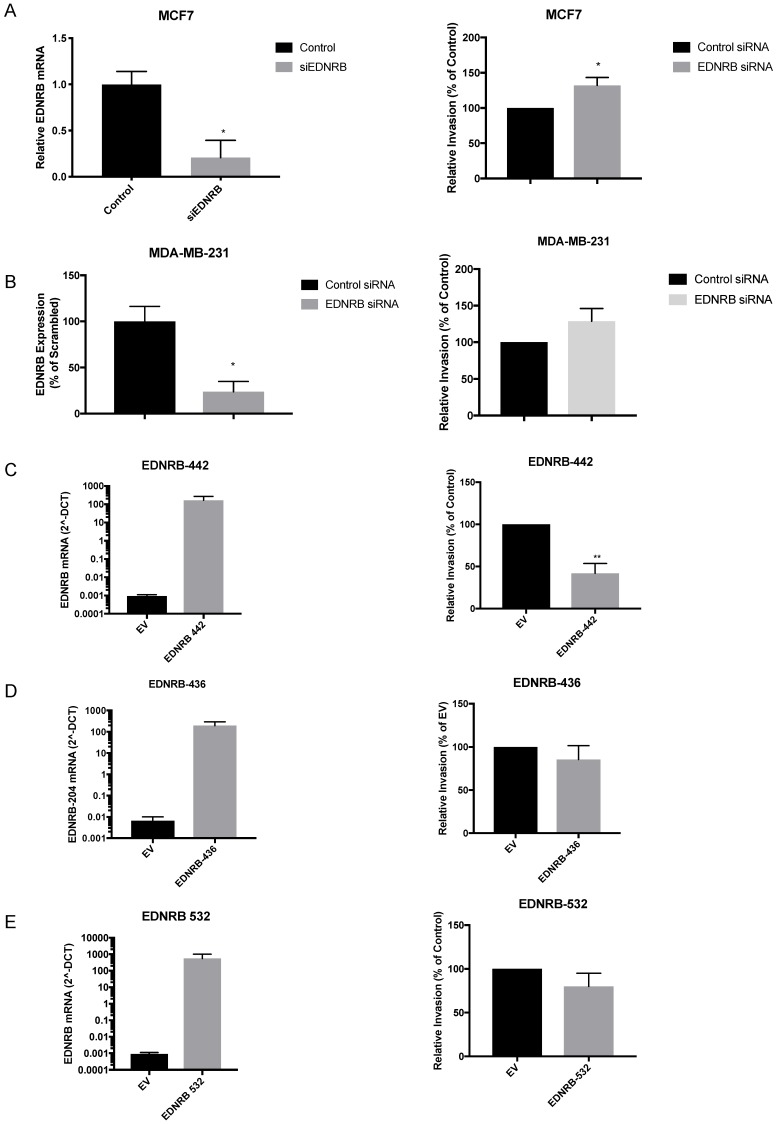
** EDNRB isoforms differentially regulate breast cancer invasion.** Transfecting siRNA specific to EDNRB alongside control siRNA into breast cancer cell lines results in (A) significant reduction in mRNA expression (p=0.027) and a significant increase in *in vitro* invasion toward ET3 (p=0.02) and (B) significant reduction in mRNA expression (p=0.018) but a non-significant increase in invasion toward ET3 (p=0.18) in MDA-MB-231 cells. Over-expressing mammalian plasmids encoding for (C) EDNRB-442 significantly increased mRNA expression (p=.0002) and significantly decreased *in vitro* invasion toward ET3 (p=0.008); however, expressing EDNRB isoforms EDNRB-436 (D) and EDNRB-532 (E) significantly increased expression (p=0.0002; p=0.0026) but did not alter invasion toward ET3 (p=0.39; p=0.25, respectively). All graphs are from 3 biological replicates.

**Figure 4:**
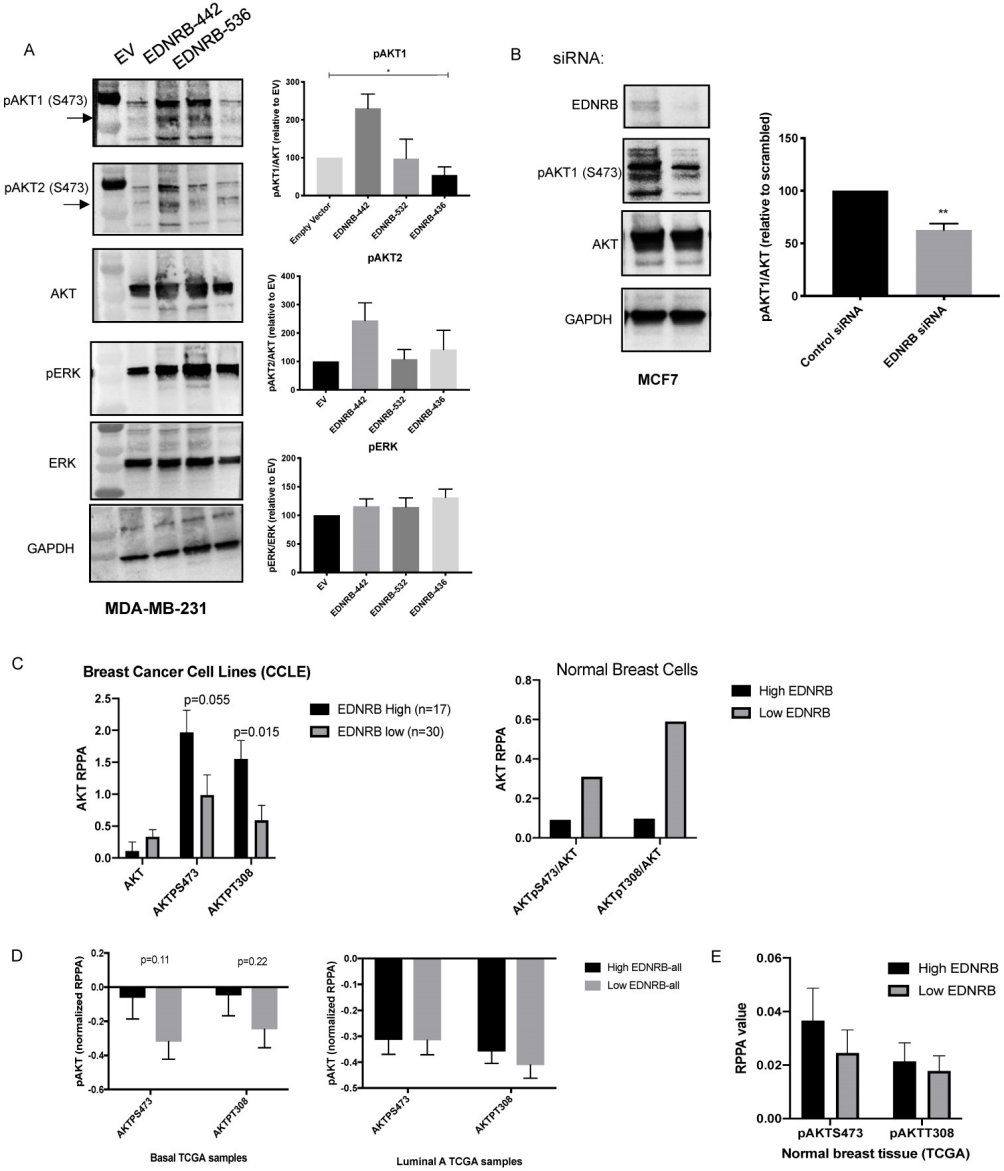
** EDNRB isoforms differentially regulate cellular signaling.** Western blotting of lysates extracted from (A) MDA-MB-231 cells transfected with EDNRB isoforms and (B) MCF-7 cells transfected EDNRB-specific siRNA were probed with antibodies specific for phosphorylated AKT1 and phosphorylated AKT2 at S473 (pAKT1, pAKT2), pan AKT, phosphorylated ERK (pERK), pan ERK, and GAPDH. Bands were quantitated using ImageJ Software and normalized to GAPDH intensity. (A) MDA-MB-231 cells transfected with EDNRB-442 had significantly higher levels of pAKT1 (ANOVA p=0.013) but not pAKT2 (p=0.28) or pERK (p=0.42); (B) MCF-7 cells transfected with EDNRB-siRNA had significantly reduced pAKT1/AKT levels (p=0.097). (C) Analysis of breast cancer cell lines from Cancer Cell Line Encyclopedia data (CCLE) shows a non-significant positive association between EDNRB and active AKT (left); this trend was not observed in non-transformed breast cell lines (right). (D) TCGA breast cancer exon expression data was separated by subtype and analyzed by median EDNRB expression using two isoform-specific probes that recognize EDNRB-442 or EDNRB-532; pAKT levels at both S473 and T308 sites from the RPPA dataset were compared between EDNRB-high and low groups (n=60 for both groups) in basal and (left) luminal A (right) breast cancer subtypes, and (E) normal breast tissue.

Although a previous study found that over-expressing and knocking down EDNRB in two breast cancer cell lines altered invasion toward endothelin-1 (ET1) 16, EDNRB-regulated invasion toward ET3 and isoform-specific effects on breast cancer invasion have not been widely studied. We first transfected MCF-7 cells with siRNA specific to EDNRB and measured its effects on invasion. Efficient knock-down of EDNRB mRNA was confirmed by RT-qPCR and Western blot (Fig. 3A, Fig. 4B); both EDNRB-442 and EDNRB-536 were similarly knocked down by the EDNRB siRNA (Supplementary Fig. S3). We next measured the effects of EDNRB knock-down on invasion toward the EDNRB-specific ligand ET3 using *in vitro* invasion assays; EDNRB knock-down significantly increased invasion in MCF-7 cells (Fig. 3A). We repeated these experiments in MDA-MB-231 cells and found a similar trend of increased invasion, but without statistical significance (Fig. 3B).

Next, we asked whether EDNRB isoforms regulate invasion similarly. Transfecting expression vectors encoding for EDNRB-442, ENDRB-532, and ENDRB-436 all resulted in increased mRNA (Fig. 3C, D, E) and protein (Supplementary Fig. S4) expression. Only EDNRB-442 over-expression significantly decreased *in vitro* invasion toward ET3 in MDA-MB-231 cells (Fig. 3C); EDNRB-532 and EDNRB-436 did not alter invasion in these cells. Over-expressing EDNRB-442 in MCF-7 cells also did not significantly affect invasion toward ET3 (Supplementary Fig. S5). Interestingly, while neither EDNRB-442 nor EDNRB-436 altered cell viability as measured by MTT assays, cells transfected with EDNRB-532 had significantly higher viability 24 hours after transfection than cells transfected with empty vector controls (Supplementary Fig S6). Together with the results of the previous figure, this suggests that in the presence of ET3, EDNRB-442 negatively regulates invasion and EDNRB-532 promotes cell viability in breast cancer cells.

### EDNRB isoforms differentially regulate AKT signaling

Endothelin B receptor signaling activates G-proteins that can stimulate (PI3K) and the mitogen activated protein kinase (MAPK) pathways 1. To determine whether EDNRB isoforms differentially regulate cell signaling, we measured activation of AKT and ERK, the primary mediators of PI3K signaling and MAPK signaling, respectively. Because AKT1 and AKT2 have been shown to differentially regulate invasion and proliferation in breast cancer 35,36, we used isoform-specific antibodies to measure AKT1 and AKT2 phosphorylation at S473. Activation of all AKT isoforms at T308 was also measured with a non-isoform specific AKT antibody, and no differences between EDNRB isoforms were observed (data not shown). We stimulated cells with ET3 24 hours after transfecting MDA-MB-231 cells with each of the three major EDNRB isoforms and found that while EDNRB-442 significantly increased pAKT1/AKT ratios, the other EDNRB isoforms did not significantly change AKT1 activation (Fig. 4A). This observation is consistent with the described role of AKT1 negatively regulating breast cancer cell invasion 37,36,35. We observed a similar trend using the pAKT2 antibody, though the differences were not statistically significant. Additionally, we did not notice any changes in ERK signaling between the EDNRB isoforms, suggesting that PI3K/AKT signaling may be the primary pathway differentially regulated by EDNRB isoforms (Fig. 4A). While we did not observe any changes in AKT1 activation in MCF-7 cells transfected with EDNRB (Supplementary Fig. S7A), this could be explained by the endogenously high EDNRB expression in these cells.

To determine whether EDNRB knockdown has similar effects on AKT signaling, we tested AKT1 phosphorylation in MCF-7 cells transfected with EDNRB siRNA. Loss of EDNRB in MCF-7 cells led to a decrease in pAKT1 in cells stimulated with ET3 (Fig. 4B). EDNRB knockdown in MDA-MB-231 cells did not significantly alter AKT1 activation (Supplementary Fig. 7B); in light of the previous results with EDNRB-442 expression in MDA-MB-231 cells, this finding is likely due to the low endogenous EDNRB expression in these cells. To determine whether this trend exists across numerous breast cancer cell lines, we analyzed data from the Cancer Cell Line Encyclopedia (CCLE); sorting breast cancer cell lines by EDNRB RNASeq values and analyzing protein expression by RPPA showed a similar trend of increased AKT activation (Fig. 4C); AKT analysis of the 2 available non-cancerous breast cell lines from CCLE by EDNRB expression suggest an opposite pattern, where high EDNRB is associated with lower AKT activation (Fig. 4C). Interestingly, we observed a non-significant positive association between EDNRB and pAKT in cancer cell lines derived from human kidney cancer, but not liver and ovarian-derived cancer cell lines (Supplementary Figs. S8).

Next, we asked whether patient tumor samples from The Cancer Genome Atlas (TCGA) showed an association between EDNRB mRNA expression and AKT activation. Analysis of breast cancer samples from TCGA revealed a non-significant trend of increased pAKT at both S743 and T308 in EDNRB-high breast cancer samples from basal, but not luminal subtypes or uninvolved breast tissue (Fig. 4D-E). We next asked whether EDNRB isoforms might also regulate AKT activation in other cancers. We analyzed EDNRB exon expression data and RPPA data from The Cancer Genome Atlas for both renal cell carcinoma and ovarian cancer. In renal cell carcinoma, both EDNRB-442 and EDNRB-532 are associated with tumors with increased pAKT at S473, while in ovarian cancer, there was only a slight association between EDNRB-442 and pAKT_S473 (Supplementary Fig. S9). Together with the previous results, we conclude that EDNRB, particularly EDNRB-442, regulates AKT1 activation in some, but not all, breast cancer cells, and that this effect may also be present in other cancer types such as kidney cancer.

### Endothelin receptors as predictors of breast cancer prognosis

**Figure 5:**
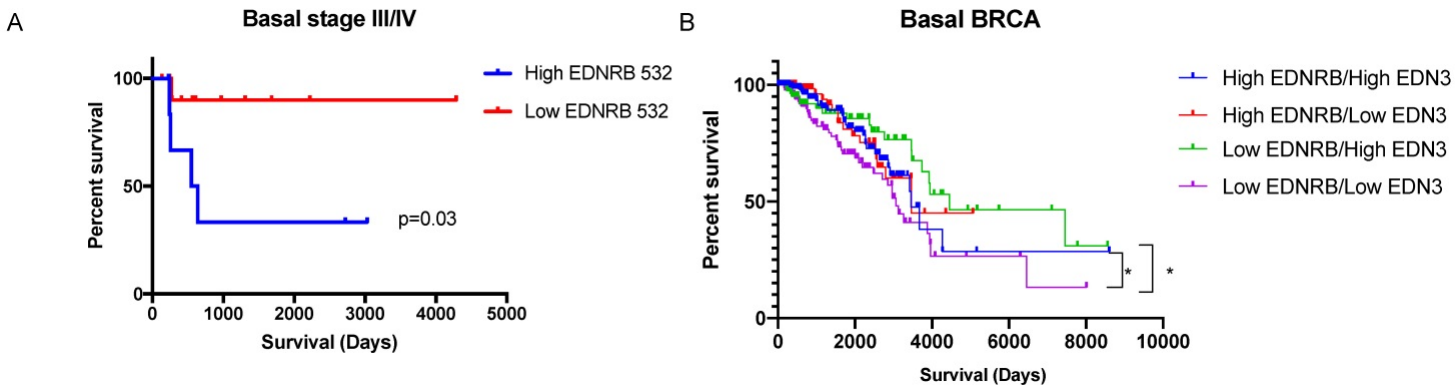
** EDNRB isoform and ligand expression may predict survival outcomes in basal breast cancers.** Survival data from the TCGA dataset was sorted based on average EDNRB survival by exon-specific probe. Basal breast cancers high in EDNRB-532 (n=7) had significantly poorer survival outcomes than basal breast cancers low in EDNRB-532 (n=12) (p=0.03, Student's t-test). (B) Sorting basal breast cancers by combined EDNRB-all and EDN3 RNASeq data showed a significant difference in survival between cancers sorted by low EDNRB/high EDN3 (n=89) vs low EDNRB/low EDN3 expression (n=158) (p=0.013).

We next asked whether EDNRB mRNA expression correlates with breast cancer patient survival. To address this, we analyzed RNAseq gene expression data from The Cancer Genome Atlas Breast Cancer dataset (TCGA BRCA) and from the exon expression RNAseq dataset (xenabrowser.net). Patient data was first separated by breast cancer subtype then dichotomized by average EDNRB RNASeq expression values. EDNRB mRNA values from the gene expression data (EDNRB-all) did not significantly predict survival across any subtype (Table 1). However, analyzing isoform-specific EDNRB expression from the exon expression dataset showed that high EDNRB-532 predicted a significantly poorer outcome in stage III/IV basal breast cancers (Table 1, Fig. 5A; HR 7.7). Interestingly, EDNRB-436 trended toward protection in this same cancer subtype and stage, though the difference was not significant (Table 1). Analysis of ovarian and liver cancer datasets with EDNRB exon expression revealed no differences in survival. In contrast to our findings in breast cancer, analysis of renal cell carcinoma revealed that EDNRB-532 expression was associated with significantly improved survival outcomes (Supplementary Fig. S10). These data reveal that the EDNRB isoform association with clinical outcome is likely cancer type-specific.

Because the downstream effects of EDNRB are likely affected by the presence of its major ligands, ET1 and ET3 (encoded for by EDN1 and EDN3, respectively), we also analyzed survival in breast cancer subtypes by relative combined expression of EDNRB/EDN3 and EDNRB/EDN1 mRNA. No significant differences in survival were observed in either basal or estrogen receptor-positive (ER+) breast cancers when stratified by EDNRB/EDN1 ratio. However, patients with low EDNRB expression had significantly higher survival if they also had high EDN3 mRNA than patients with low EDNRB and low EDN3 levels (Fig. 5B). Likewise, patients with high EDNRB and high EDN3 levels had better outcomes than patients with low EDNRB and low EDN3 levels (Fig. 5B). Together, these results suggest that EDNRB isoforms have differential potentials as biomarkers, that EDNRB-536 may be a specific biomarker for estrogen-receptor negative and triple-negative breast cancers, and that the utility of EDNRB as a potential biomarker may depend on the expression level of EDN3.

## Discussion

While there are numerous reports characterizing EDNRB expression in breast cancer, our research adds to the knowledge of endothelin B receptor (EDNRB) in breast cancer by showing differences in both the expression and function of specific EDNRB isoforms in breast cancer cells. Specifically, we found that EDNRB isoform expression differed amongst breast cancer cell lines and between normal and cancer cells. Furthermore, we found that the EDNRB-442 isoform negatively regulates invasion toward ET3, while the other major isoforms have no significant effect on invasion. Consistently, our data show a EDNRB-442 induced increase in ET3-stimulated pAKT1 signaling in breast cancer cell lines, while none of the EDNRB isoforms altered ERK activation, suggesting that ET3/EDNRB signaling primarily functions through the PI3K pathway. Finally, we observed a EDNRB-532 dependent increase in cell viability and a decrease in survival in stage III/IV basal breast cancer patients with high EDNRB-532, but no other isoforms or cancer subtypes showed significant differences in survival. Together, our results provide some explanation of the previous contradictory results regarding the endothelin B receptor in breast cancer and support a more complex model of the involvement of EDNRB in its regulation of breast cancer progression that is isoform, ligand, and subtype specific.

The literature is somewhat conflicted regarding whether EDNRB expression and activity in breast cancer cells promotes breast cancer or inhibits its progression. Because EDNRB has been proposed to serve as a negative regulator of endothelin A receptor signaling (EDNRA) 5,9, and considering that EDNRA is a well-known promoter of breast cancer progression 19, EDNRB is expected to be an antagonist to breast cancer. However, most of the studies published on EDNRB in breast cancer characterize it as a cancer promoter 12,14-16,20. The reason for this discrepancy is unclear from previous studies. We designed the present study to help clarify how EDNRB regulates breast cancer progression by introducing two novel components. First, we focused on the EDNRB-selective ligand endothelin-3 (ET3) rather than ET1 throughout our studies. This allowed us to isolate EDNRB function from endogenous EDNRA activity and specifically study the effects of ET3-activated EDNRB signaling. Second, by studying specific isoforms of EDNRB we also describe distinct roles for the EDNRB-442 and EDNRB-532 isoforms that may explain some of the conflicting reports regarding the contribution of EDNRB to breast cancer progression.

When comparing EDNRB mRNA and protein expression in breast cancer cells, we found that the MCF-7 cancer cell line had the highest EDNRB expression of the cell lines tested, while normal breast epithelial cells (HMECs) did not express detectable levels of EDNRB (Fig. 1B, Fig. 2). In contrast to these findings, Hagemann, et al reported that MDA-MB-231 cells had the higher EDNRB mRNA expression than MCF-7 cells 16. One possible explanation for the differences in their study and our study is the cell culture conditions used to grow cells. Hagemann et al. report using insulin in all of their cultures, while insulin was removed from our cells prior to RNA and protein extraction; thus the results shown here may be a more accurate representation of the inherent differences in EDNRB expression between these cell lines independent of exogenous insulin. Unlike the data from cultured primary breast cells, normal/uninvolved breast tissue expressed significantly higher EDNRB than the breast tumor samples (Fig. 1G), suggesting that stromal cells may contribute to the EDNRB expression observed in human tissue samples. Similarly, we found isoform expression differences in breast tissue and human breast cancer samples compared to our cell line analyses; in the tissue samples, EDNRB-532 appeared to be the predominant isoform, while EDNRB-442 was primarily expressed in cancer cell lines. Interestingly, we did observe that low-grade cancers expressed EDNRB-442 predominantly, while high grade cancers mainly expressed EDNRB-532 (Fig. 1G). More research is needed to clarify these differences.

The PI3K/AKT pathway is a well-established oncogenic pathway, and AKT1 and AKT2 have repeatedly been shown to differentially regulate breast cancer cell proliferation and invasion. Specifically, while AKT2 activates epithelial mesenchymal transition and facilitates breast cancer cell invasion and metastasis, AKT1 negatively regulates AKT2-induced EMT and invasion 37, 36, 35. Thus, our observations that EDNRB-442 activates pAKT1 and abrogates breast cancer invasion are consistent with previous studies describing AKT1 as a negative regulator of breast cancer. While our data generally supports that EDNRB-442 abrogates invasion toward ET3 and activates AKT1, we did observe slight cell line specific differences in these studies. Over-expressing EDNRB-442 effectively inhibited invasion (Fig. 3) and activated AKT1 (Fig. 4) in the low-EDNRB expressing MDA-MB-231 cell line, but these effects were not significant in the MCF-7 cells (Supplementary Fig. S4, S6). Likewise, our knockdown studies showed more significance in the high-EDNRB expressing MCF-7 cell line than the MDA-MB-231 cells. Because we found significant effects in both cell lines, we believe the most likely explanation for the discrepancies is that over-expression is more likely to produce significant results in cell lines with lower endogenous EDNRB expression, and knockdown is more likely to produce notably effects in cell lines with high endogenous EDNRB expression. Thus, we believe our data support a general effect of EDNRB-442 on breast cancer cell invasion and AKT1 activation across subtypes.

The results of our ET-3 stimulated invasion assays reveal an inhibitory role of ET3-stimulated EDNRB on breast cancer invasion (Fig. 4), in contrast to the pro-invasive role of ET1-stimulated EDNRB reported by Hagemann et. al. 16. These differences suggest ligand-specific effects of EDNRB on cell invasion. In support of this, we did not observe significant differences in invasion in EDNRB-knockdown cells in the absence of ET3 (unpublished data), suggesting the importance of ET3 in regulating EDNRB-dependent invasion. Because AKT1 is a known negative regulator of invasion in breast cancer cells, our findings suggest that EDNRB-442 activates AKT1 and thereby inhibits breast cancer invasion.

Our observation that in the TCGA dataset, only EDNRB-532 alters survival in basal cancers is consistent with our data showing EDNRB-532 promotes cell viability (Fig. S5). Interestingly, while EDNRB expression did not significantly predict patient outcome in liver and ovarian cancer, high EDNRB 436/442 and EDNRB-532 expression predicted improved patient outcome in renal cell carcinoma (Fig. S10), suggesting cancer type-specific effects of EDNRB on patient outcome. Somewhat surprisingly, TCGA breast cancer dataset analysis did not show significant survival differences in cancers expressing high levels of EDNRB-442. This may be because of the potential ligand-specific effects of EDNRB-442. Our finding that when combining breast cancers low in EDNRB expression with the gene coding for ET3 (EDN3) supports this finding; while no significant differences were found with combined EDNRB and EDN1 mRNA expression, we did find that basal cancers with high EDN3 and low EDNRB have better survival (Fig. 5B). Cancers with lower EDNRB expression and higher EDN3 expression would be expected to have most EDNRB receptors saturated by ET3, preventing any ET1-stimulated signaling effects. Further investigation is warranted to test this idea.

The results presented in this study may also have relevance to therapeutically targeting the endothelin receptors in breast cancer. While inactivation of the endothelin A receptor leads to decreased tumor growth in pre-clinical models 38, 39, 40, 41 and regulates epithelial-mesenchymal transition and tumor vascularization 42, 43, clinical trials targeting this receptor in prostate and ovarian cancer have been largely ineffective 44, 45. This has prompted some to propose dual targeting both endothelin receptors (EDNRA and EDNRB). While some pre-clinical studies suggest benefit with dual antagonists in breast cancer models 46, 47, 48 our results suggest that more careful targeting of these receptors and their specific isoforms may be warranted.

## Supplementary Material

Supplementary figures and tables.

## Figures and Tables

**Table 1:** Summary of Mantel-Cox survival analysis by EDNRB isoform expression.

Molecular Subtype	Stage	EDNRB-All p-value/HR	EDNRB-442/436 p-value/HR	EDNRB-532 p-value/HR
Basal	I/II	0.15/0.41	0.51/0.67	0.69/0.74
Basal	III/IV	0.39/2.04	0.98/1.02	0.03/7.7
LumA	I/II	0.63/1.18	0.52/1.3	0.62/1.2
LumA	III/IV	0.86/0.92	0.63/0.76	0.43/0.66
LumB	I/II	0.49/0.92	0.61/0.75	0.21/2.14
LumB	III/IV	0.46/1.79	0.9/1.08	0.13/0.423
HER2	I/II	0.63/0.81	0.88/0.85	0.7/0.67
HER2	III/IV	0.76/1.1	0.27/2.4	0.99/1.01

TCGA exon expression data was sorted by subtype and stage, then dichotomized based on mean EDNRB values. P-values and hazard ratios (HR) are shown for each isoform. The same exon probe detects both EDNRB-442 and EDNRB-436, so these were combined in this analysis.

## Abbreviations

EDNRB: endothelin B receptor
EDNRA: endothelin A receptor
GPCR: G-protein coupled receptor
ET1: Endothelin-1
ET2: Endothelin-2
ET3: Endothelin-3
TNBC: triple negative breast cancer
