# Supplementary material for: Differences in Endothelin B Receptor Isoforms Expression and Function in Breast Cancer Cells
Source: J Cancer. 2020 Feb 19;11(9):2688–701. doi: 10.7150/jca.41004 (PMC7066022; doi:10.7150/jca.41004)
Supplement: Supplementary file 1 — Supplementary figures and tables. [file jcav11p2688s1.pdf]

Differences in Endothelin B Receptor isoforms expression and function in breast cancer cells

Meena Halaka, et al.

Supplementary Figure/Table Legends:

Primer Name	Primer Sequence (5'-3')
**EDNRB all F**	**CAAGGACCCATCGAGATCAAG**
**EDNRB all R**	**GACCGTTTCGCATGCACTTG**
**EDNRB 436 F**	**ATGATCACCTAAAGCAGAGACG**
**EDNRB 436 R**	**GTGGCCCAGCCTTAAAGCAG**
**EDNRB 532 F:**	**CTCGGGCAACTACTACTGATG**
**EDNRB 532 R:**	**CATCCCTTCCCATCAATCACC**
**EDNRB 532/409 F**	**GATCCGAGAGGCTCTGAAAC**
**EDNRB 532/409 R**	**GTGGGTGGCGTCATTATCTC**
**ACTB F**	**AGAGCTACGAGCTGCCTGAC**
**ACTB R**	**AGCACTGTGTTGGCGTACAG**

Supplementary Table 1. Primer Sequences used in this study are shown.

	EDNRB-532	EDNRB-442	EDNRB-436	Start	End	Exon Probe in TCGA dataset
**Exon 1a (372 bps)**	**X**			**60,691**	**61,063**	**Probe 8 (chr13:78493532-78493903:-)**
**Exon 1b (742 bps)**		**X** ***Non-coding; full exon is present only in 1 EDNRB-442 splice variant***		**61,698**	**62,439**	**Probe 7 (chr13:78492226-78492966:-)**
**Exon 2 (534 bps)**	**X**	**X**	**X**	**61,906**	**62,439**	**N/A**
**Exon 3 (113 bps)**	**X**	**X**	**X**	**76,923**	**77,035**	**Probe 6 (chr13:78477630-78477742:-)**
**Exon 4 (205 bps)**	**X**	**X**	**X**	**77,170**	**77,374**	**Probe 5 (chr13:78477291-78477495:-)**
**Exon 5 (150 bps)**	**X**	**X**	**X**	**79,323**	**79,472**	**Probe 4 (chr13:78475193-78475342:-)**
**Exon 6 (134 bps)**	**X**	**X**	**X**	**79,876**	**80,009**	**Probe 3 (chr13:78474656-78474789:-)**
**Exon 7 (109 bps)**	**X**	**X**	**X**	**80,563**	**80,671**	**Probe 2 (chr13:78473994-78474102:-)**
**Exon 8 (2854 bps)**	**X**	**X**		**82,196**	**85,049**	**Probe 1 (chr13:78469616-78472469:-)**
**Exon 9 (151 bps)**			**X**	**83,971**	**84,121**	**N/A**

Supplementary Table 2. EDNRB isoform and TCGA probe chart. Shown are the exons present in each of the major EDNRB isoforms. "X" marks which exons are detected by which TCGA exon expression probes. Probe 7 (Exon 1b) detects one of the splice variants encoding for the EDNRB-442 isoform, but as this exon is non-coding and is spliced out of other EDNRB-442 isoforms, it does not represent expression of all EDNRB-442 variants.

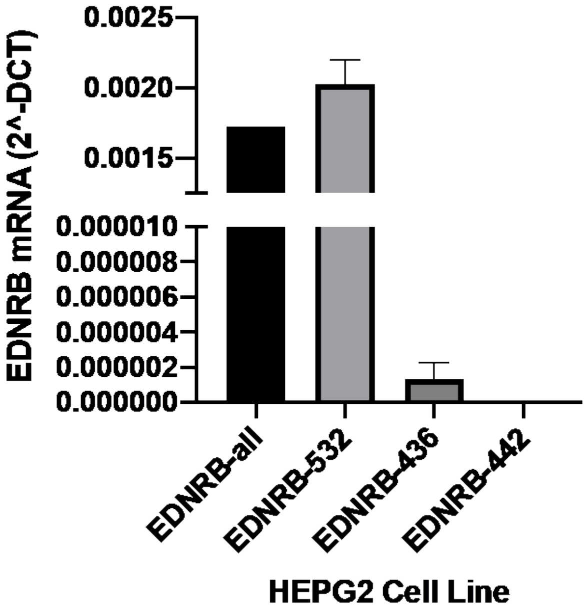

Supplementary Figure S1. HEPG2 expression of EDNRB isoforms. RNA isolated from HEPG2 liver carcinoma cells was tested by RT-qPCR for EDNRB isoform expression. Note that overall EDNRB expression is lower in HEPG2 cells compared to most of the tested breast cancer cell lines; furthermore, EDNRB-532 appears to the predominant isoform in these cells.

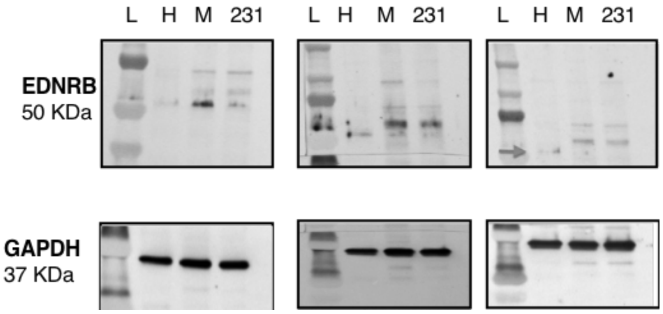

Supplementary Figure S2. EDNRB Western blots of cell lines using alternate antibody. A EDNRB-specific antibody (Santa Cruz H-74) that detects all major full-length isoforms of EDNRB was used to probe lysates from HMEC, MCF-7, and MDA-MB-231 cells. Similar expression was observed with this antibody as seen with the EDNRB-442/532 antibody shown in Figure 2.

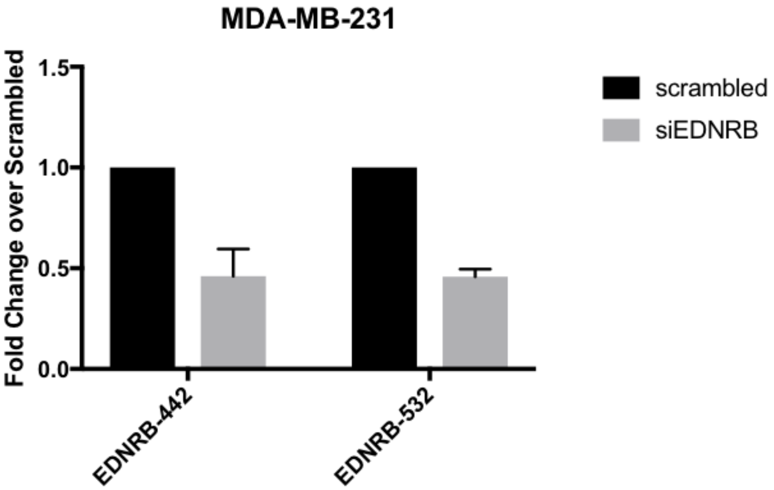

Supplementary Figure S3. Knock-down of both EDNRB-442 and EDNRB-532 with EDNRB-siRNA. MDA-MB-231 cells transfected with siRNA specific to EDNRB showed decreased levels of both EDNRB-442 and 532 variants.

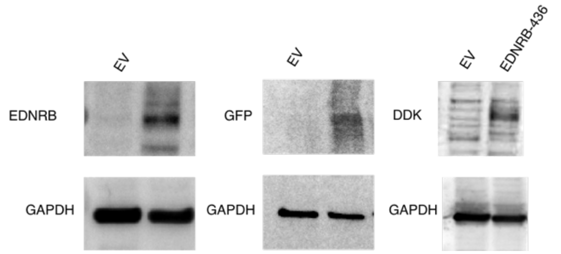

Supplementary Figure S4. EDNRB over-expression in plasmid-transfected cells. MDA-MB-231 cells transfected with plasmids expressing EDNRB-442, EDNRB-532, and EDNRB-436 were probed with antibodies specific for EDNRB, GFP, and DDK, respectively. These blots verify efficient protein expression of all three plasmids.

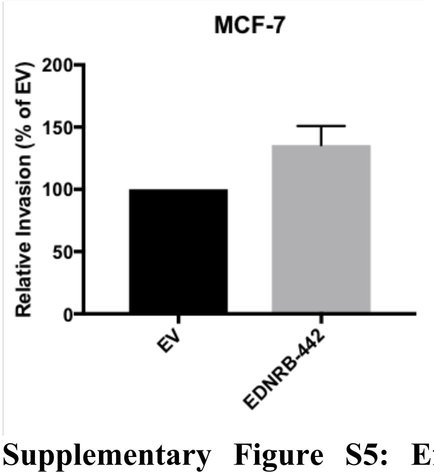

Supplementary Figure S5: Effect of EDNRB-442 on MCF-7 invasion toward ET3. Transfecting EDNRB-442 resulted in a non-significant trend of increased invasion in MCF-7 cells (p=0.08) over 3 biological replicates.

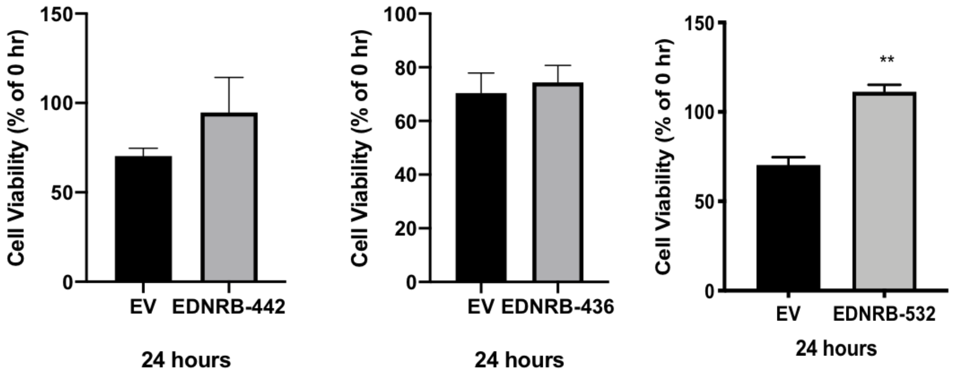

Supplementary Figure S6: EDNRB-532 alters cell viability. MDA-MB-231 cells transfected with EDNRB-442, 436, or 532 were measured by MTT assay 24 hours after transfection. Over 3 biological replicates, only EDNRB-532 significantly increased cell viability over empty vector controls (Student's t-test p=0.002).

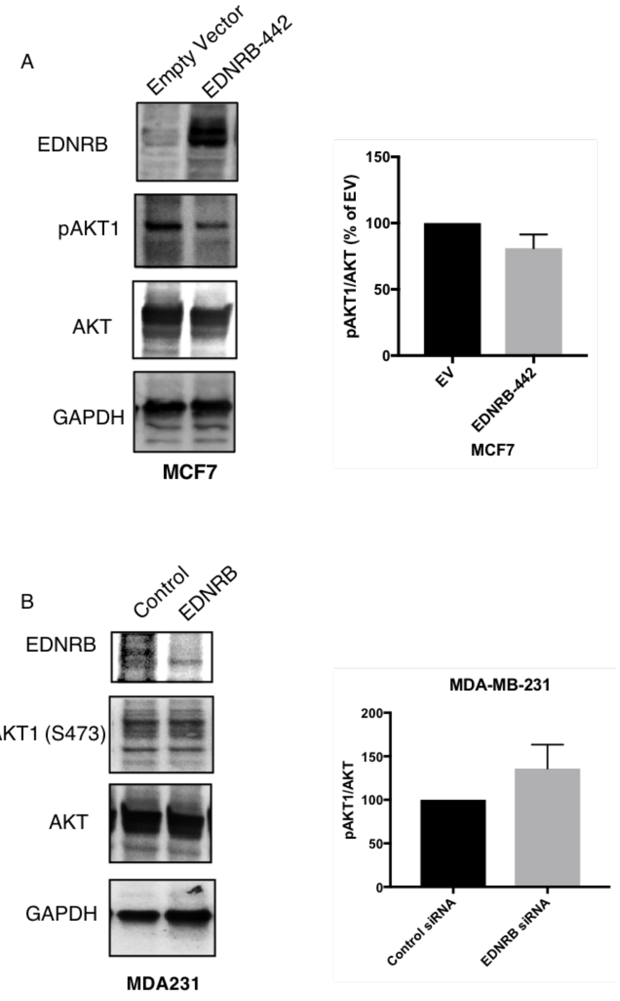

Supplementary Figure S7: EDNRB effects on AKT signaling in MCF7 and MDA-MB-231 cells. (A) MCF-7 cells transfected with EDNRB-442 did not show significant changes in pAKT1 levels when normalized to AKT over 3 biological replicates (p=0.1, Student's t-test). (B) MDA-MB-231 cells transfected with EDNRB-siRNA showed a non-significant trend of increased pAKT1/AKT (p=0.26, Student's t-test).

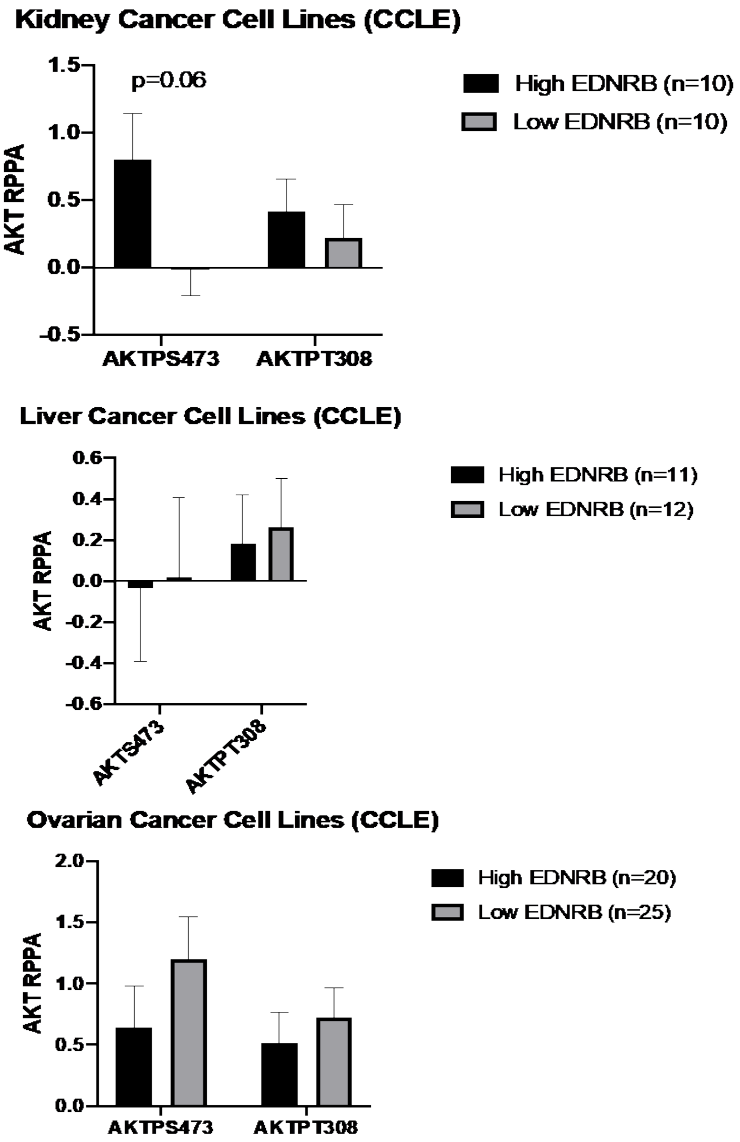

Supplementary Figure S8. Association between EDNRB mRNA and AKT phosphorylation in non-breast cancer cell lines. Kidney, liver, and ovarian cancer cell lines from the Cancer Cell Line Encyclopedia (CCLE) were sorted based on median EDNRB expression and analyzed for AKT phosphorylation at S473 and T308. Only kidney cancer cell lines approached significance with the pAKT_S473 probe (p=0.06).

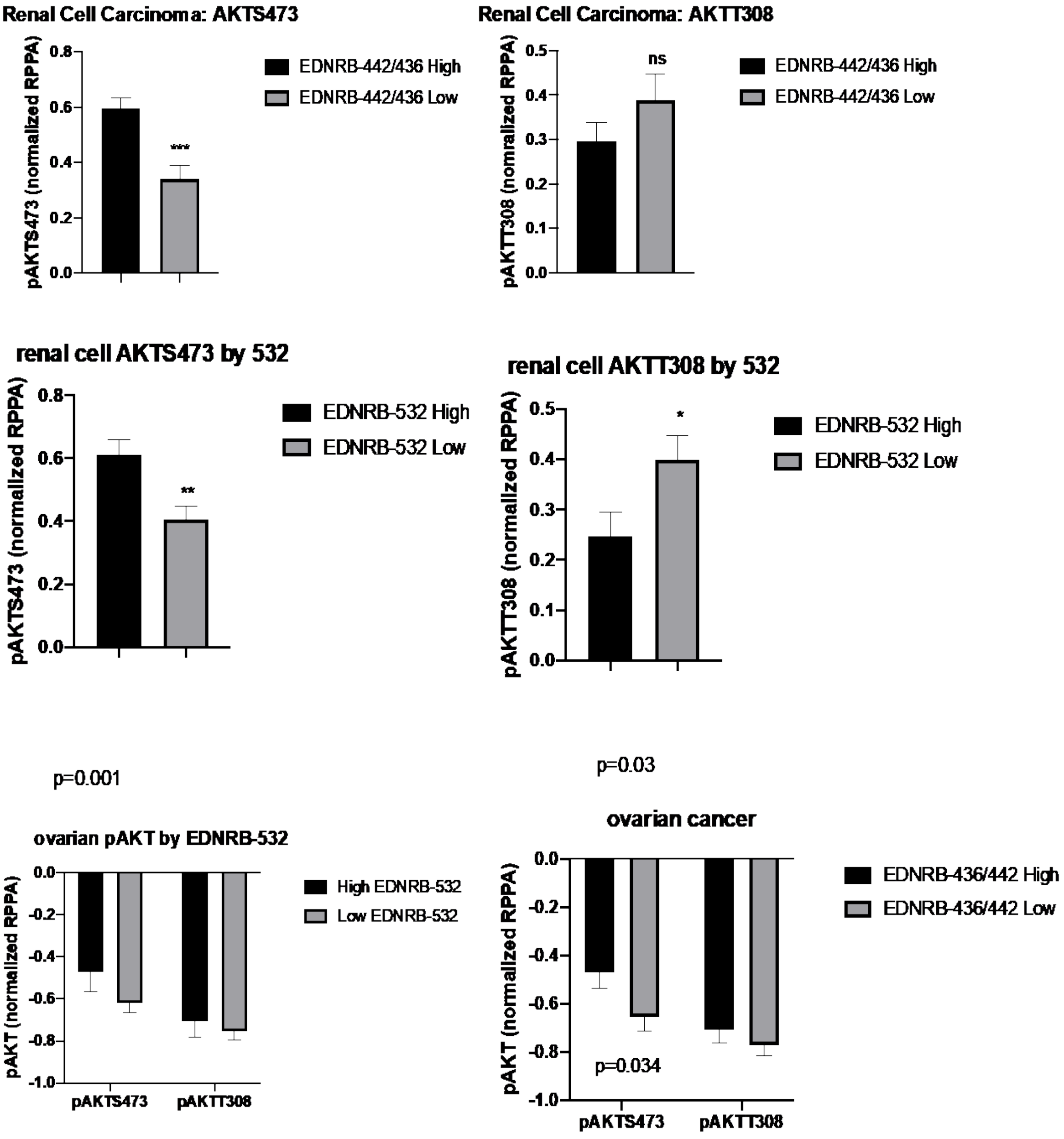

Supplementary Figure S9. Association between EDNRB isoform expression and AKT activation in non-breast human tumor samples. Data from The Cancer Genome Atlas databases from renal cell carcinoma and ovarian cancer was sorted based on median EDNRB isoform expression and analyzed for differences in AKT phosphorylation at S473 and T308. Only renal cell carcinomas showed significant differences (*** $p<0.001$; ** $p=0.001$; * $p=0.03$) between high- and low-EDNRB isoform expression and AKT activation.

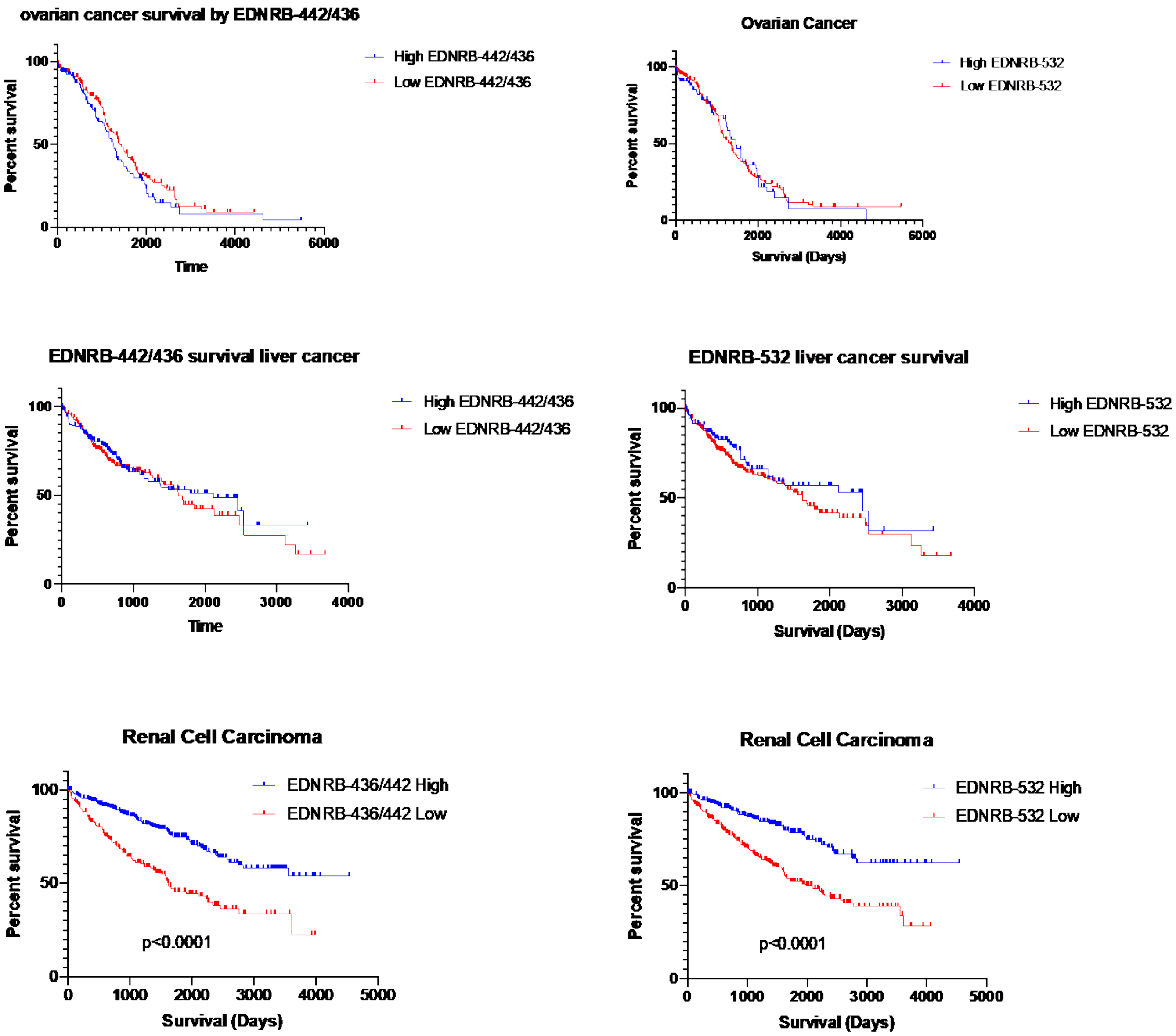

Supplementary Figure S10. Cancer patient survival based on EDNRB isoform expression. Patient survival data from The Cancer Genome Atlas was compared between patients sorted by median EDNRB isoform expression. Only renal cell cancer showed significant survival differences based on EDNRB-436/442 and EDNRB-532 expression.
